# Use of Metagenomics and Isolation of Actinobacteria in Brazil's Atlantic Rainforest Soil for Antimicrobial Prospecting

**DOI:** 10.1155/2014/909601

**Published:** 2014-03-12

**Authors:** Danyelle Alves Martins Assis, Rachel Passos Rezende, João Carlos Teixeira Dias

**Affiliations:** Laboratório de Monitoramento Ambiental/Laboratório de Biotecnologia de Microrganismos, Centro de Biotecnologia e Genética, Universidade Estadual de Santa Cruz (UESC), 45662-900 Ilhéus, BA, Brazil

## Abstract

Modern techniques involving molecular biology, such as metagenomics, have the advantage of exploiting a higher number of microorganisms; however, classic isolation and culture methods used to obtain antimicrobials continue to be promising, especially in the isolation of Actinobacteria, which are responsible for the production of many of these compounds. In this work, two methodologies were used to search for antimicrobial substances—isolation of Actinobacteria and metagenomics of the Atlantic Rainforest soil and of the cultivation of cocoa intercropped with acai berry in the Atlantic Rainforest. The metagenomic libraries were constructed with the CopyControl Fosmid Library kit EPICENTRE, resulting in a total of 2688 clones, 1344 of each soil sample. None of the clones presented antimicrobial activity against the microorganisms tested: *S. aureus*, *Bacillus subtilis,* and *Salmonella choleraesuis*. A total of 46 isolates were obtained from the isolation of soil Actinobacteria: 24 isolates from Atlantic Rainforest soil and 22 isolates from the intercrop cultivation soil. Of these, two Atlantic Rainforest soil isolates inhibited the growth of *S. aureus* including a clinical isolate of *S. aureus* MRSA—a promising result, since it is an important multidrug-resistant human pathogen.

## 1. Introduction

The search for new antimicrobials becomes more important because of the increasing resistance of pathogenic microorganisms. However, the decrease of the development of the antibacterial drugs has been reported, due to the abandonment and reduction of research and difficulty in isolating novel compounds which can cope with resistant pathogens [1–3].

Over two-thirds of clinically used antibiotics consist of natural products or their semisynthetic byproducts [4], and the majority of known natural products with antimicrobial activity are from soil Actinobacteria [5]. This group of bacteria is very varied physiologically and metabolically and forms important substances, such as enzymes and powerful antibiotics [6–8].

In recent years, researchers have turned to untapped natural sources and to places with extreme environmental conditions of temperature, pH, and humidity, believing that organisms obtained from ecosystems that have not yet been exploited are frequently associated with new physiological and metabolic diversity [9]. Although soil has been exploited by the drug industry for some 50 years, only a minuscule fraction of the globe has been sampled, and only a small fraction of Actinobacteria has been discovered [10].

There has been a lot of discussion about the fact that only 1% of the microbial diversity is accessible to traditional cultivation techniques [11] and that 99% of microorganisms unsuitable for growth can provide an unexploited wealth of new antibiotics and other secondary metabolites [12]. This statement is based on the use of metagenomics, which enables the exploitation of organisms using techniques that do not depend on cultivation, by accessing their genetic material [12]. Metagenomics involves the direct extraction of DNA from the environment sample, cloning it in an appropriate vector, and inserting it in bacteria that can be cultivated for analysis of all genomes from a certain environment, therefore providing access to all microbial communities, both to cultivable microorganisms and for those uncultured [13].

Major natural resources (such as forests and unique environmental habitats) are mainly located in developing countries [14]. Such is the case of the Atlantic Rainforest, an ecosystem largely located within Brazilian territory that has a high level of biological diversity and enormous potential as a virtually unexploited source of new molecules, such as antimicrobials and enzymes [15–17]. Protected areas holding the last large remains of Atlantic Rainforest in Brazil are the cocoa growing region of southern Bahia and the Serra do Mar mountain range that stretches through Rio de Janeiro, São Paulo, and Paraná [18].

Some authors note that the isolation and selection of microorganisms or their genes from a variety of environments using a variety of tracing methods will provide numerous opportunities to discover new drugs and other products [19]. In this work, we used two approaches to search for antimicrobials, a modern technique with the construction and classification of two metagenomic libraries with fosmid vectors and a classic technique, with the isolation of Actinobacteria from the Atlantic Rainforest soil and from an area planted with cocoa intercropped with acai berry in the Atlantic Rainforest.

## 2. Materials and Methods

### 2.1. Microorganisms and Cultivating Conditions

The standard microorganism strains used in the antimicrobial assays were* Staphylococcus aureus* (CCMB 262),* Salmonella choleraesius* (CCMB 281) and* E. coli* (CCMB 261) from the Coleção de Culturas de Microrganismos da Bahia (CCMB)-UEFS, Feira de Santana, Bahia, Brazil;* Staphylococcus saprophyticus* (ATCC 35552) and* Shigella flexneri* (ATCC 12022) from the American Type Culture Collection (ATCC), Manassas, USA;* Bacillus subtilis* (INCQS 00002) from the Instituto Nacional de Controle de Qualidade em Saúde (INCQS) Fiocruz, Brazil; and a clinical isolate of methicillin-resistant* S. aureus*, obtained from volunteers at a hospital in Itabuna, Bahia, Brazil. The microorganisms were cultivated in Luria-Bertani-LB (Himedia) medium at 37°C for 16 hours. The inoculums were standardized according to a McFarland scale 0.5.

### 2.2. Obtaining Soil Samples

Atlantic Rainforest soil samples were collected from the Una Bahia EcoPark, Brazil, location 15°11′36.8′′ S, 39°01′49.6′′ W, elevation 17 m. Soil samples from the area planted with cocoa intercropped with acai berry in the Atlantic Rainforest were collected in the same municipal area, at a location near the Una Biological Reserve, location 15°10′47.7′′ S, 39°02′49.9′′ W, elevation 20 m. Five samples were collected in areas of 1 m^2^ with depth 0–10 cm.

### 2.3. DNA Extraction and Construction of Metagenomic Libraries

High molecular weight DNA from both soils was obtained by extraction with PowerMax Soil DNA Isolation kit (MoBIO, USA) according to the manufacturer's instructions. The metagenomic libraries were constructed with the cloning kit CopyControl Fosmid Library EPICENTRE-pCC1FOS, which has fosmid vectors and* E. coli* host cell EPI300 following the manufacturer's instructions. DNA was purified by electrophoresis on 1% low melting point agarose and the DNA between 35 and 40 kb was excised and extracted from the gel with Gelase (EPICENTRE). The selected DNA was ligated to the Copy Control pCC1FOS vector (EPICENTRE). Lambda Packaging Extracts (MaxPlax EPICENTRE) were added to the ligation mixture, and infection of cells of* E. coli* EPI300 was performed according to the manufacturer's instructions.* E. coli* transformants grown in LB with chloramphenicol 12.5 *μ*g/mL at 37°C for 16 hours were transferred individually to 96-well plates and maintained in LB medium with 20% glycerol at −80°C. One metagenomic library was constructed for each soil sample (Atlantic Rainforest and cocoa intercropped with acai berry in Atlantic Rainforest).

### 2.4. Antimicrobial Testing of Metagenomic Libraries

Assays for bioprospecting of antimicrobial compounds from metagenomic libraries were performed on Müeller Hinton agar (Merck), pH 7,2 (±0,2), with 0.2 mL of the standardized inoculum of microorganisms:* S. aureus* (CCMB 262),* Salmonella choleraesius* (CCMB 281), and* Bacillus subtilis* (INCQS 00002). The clones contained in the 96-well plates were inoculated with a replicator, and the plates were incubated at 37°C for 16 hours.

### 2.5. Isolation of Actinobacteria from Soil Samples

Serial dilutions (10^−1^ to 10^−7^) of the soil samples were made in NaCl 0.85% sterile solution. An aliquot of 0.1 mL of each dilution was inoculated in dishes containing Caseinate-Dextrose-Agar medium (glucose 2 g/L, casein 0.2 g/L, MgSO_4_ 0.2 g/L; K_2_HPO_4_ 0.5 g/L; FeCl_3_ 0.005% and agar 15 g/L) [20], and incubated at 28°C for up to 21 days. The colonies were picked to Petri dishes containing* International Streptomyces Project* medium 2 (*ISP*2) [21].

### 2.6. Obtaining Actinobacteria Fermentation Broth

The isolated Actinobacteria colonies were cultivated in 50 mL of* International Streptomyces Project* 2 (*ISP*2) medium [21], at 28°C with constant agitation at 150 rpm during 14 days. 1.5 mL of the fermentation broth was transferred to 2 mL tubes and centrifuged at 5000 g for 10 minutes; 100 *μ*L of the supernatant was used for the antimicrobial activity assays.

### 2.7. Antimicrobial Assay of Actinobacteria Isolates

The antimicrobial detection assays were performed using the agar diffusion technique (well method) [22], in Müeller Hinton agar, against test microorganisms:* Staphylococcus aureus* CCMB 262,* Salmonella choleraesius* CCMB 281,* E. coli* CCMB 261,* Bacillus subtilis* INCQS 00002,* Staphylococcus saprophyticus* ATCC 35552,* Shigella flexneri* ATCC 12022, and methicillin-resistant* S. aureus *(MRSA). In wells of 6 mm of diameter made in culture medium, were inoculated separately 100 *μ*L of the supernatant obtained after growth of each isolate in ISP2 medium. The dishes were incubated for 16 hours at 37°C.

### 2.8. Antimicrobials Stability Analysis

The antimicrobials were tested in their stability and resistance to storage time, temperature, and autoclaving. To that purpose, the extracts were stored in fridge (2 to 8°C) for 10 months and in freezer (−20°C) for 10 months, and the obtained extracts were autoclaved for 15 min at 121°C and 1 atm. Inhibition tests were carried out using the agar diffusion method as previously described, with extracts of 3 different treatments.

## 3. Results

### 3.1. Obtaining Metagenomic Libraries and Antimicrobial Assay

The DNA from the soils of the Atlantic Rainforest and the intercropping planting system extracted with the PowerMax Soil DNA Isolation kit was satisfactory, because it enabled the extraction of high molecular weight DNA with the necessary fragment sizes for fosmid vector cloning, with DNA yields of 565 ng/*μ*L for the Atlantic Rainforest soil, and 498 ng/*μ*L for the intercropping planting system. We obtained 1344 clones from Atlantic Rainforest soil and 1344 clones from cocoa intercropped with acai berry in Atlantic Rainforest soil. The antimicrobial activity assays of these 2699 clones were not positive for the microorganisms tested.

### 3.2. Antimicrobial Assay of Actinobacteria Isolates

**Figure 1:**
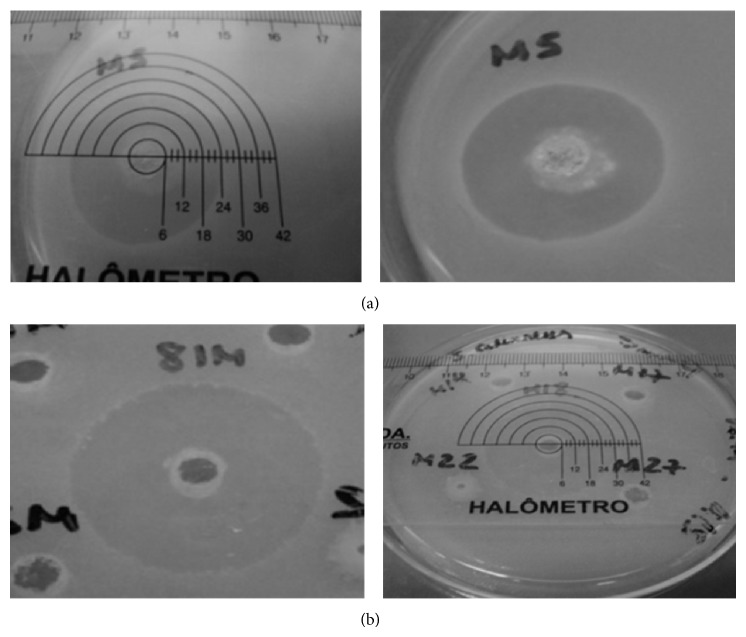
Antimicrobial assays using the agar diffusion method of isolates AR5 and AR18 against* S. aureus *CCMB 262. AR5 zone of inhibition 24 mm (a); AR18 zone of inhibition 28 mm (b).

**Figure 2:**
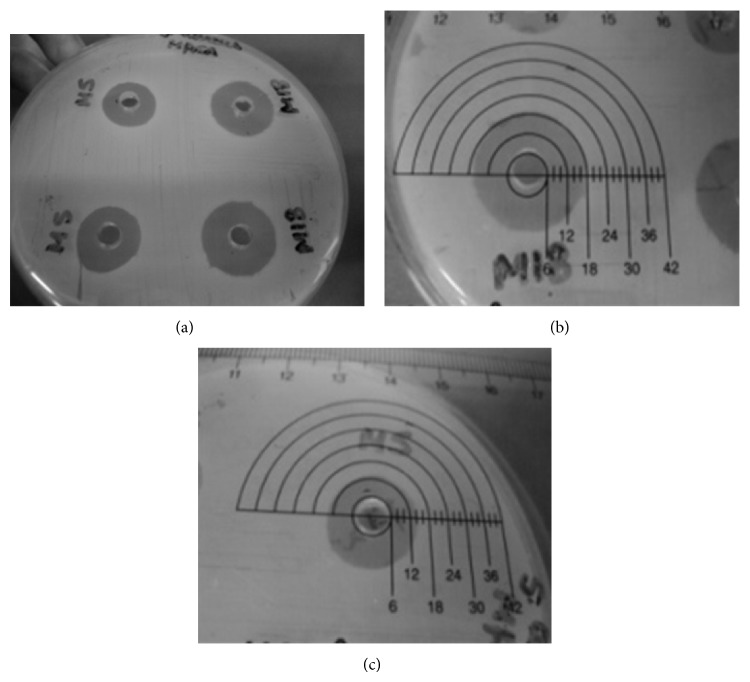
Antimicrobial assays using the agar diffusion method of isolates AR5 and AR18 against* S. aureus *MRSA (a); AR5 Zone of inhibition 14 mm (b); AR18 Zone of inhibition 18 mm (c).

A total of 24 Atlantic Rainforest soil microorganisms were isolated, as well as 22 from the intercropping system soil. Isolates 5 and 18 of the Atlantic Rainforest soil (AR5 e AR18) had positive inhibition results against* Staphylococcus aureus* CCMB 262 and for clinical* Staphylococcus aureus* MRSA clinical isolate. Isolate 5 had a 24 mm inhibition zone for* S. aureus* CCMB 262 and 16 mm for* S. aureus* MRSA; and isolate 18 had a 28 mm inhibition zone for* S. aureus* CCMB 262 and 18 mm for* S. aureus* MRSA (Figures 1 and 2 and Table 1). The isolates from the intercropping planting system showed no inhibition against the microorganisms tested.

### 3.3. Antimicrobials Stability Analysis

**Figure 3:**
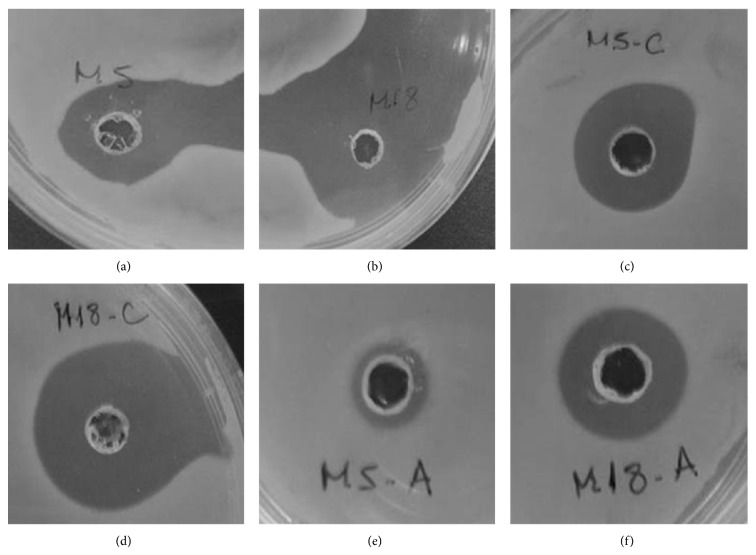
Antimicrobials assay against* S. aureus* CCMB 262 after different treatments to verify stability of the compounds. Antimicrobial AR5 stored for 10 months (2 to 8°C) (a); antimicrobial AR18 stored for10 months (2 to 8°C) (b); antimicrobial AR5 stored for 10 months (−20°C) (c); antimicrobial AR18 stored for 10 months (−20°C) (d); antimicrobial AR5 after autoclaving (121°C, 15 min, 1 atm) and (e); antimicrobial AR18 after autoclaving (121°C, 15 min, 1 atm) (f).

The antimicrobial compound produced by isolates AR5 and AR18 demonstrated stability in their antimicrobial activity in relation to storage, temperature, and autoclaving time. The antibiosis test against* S. aureus *CCMB 262 done with the antimicrobials stored for 10 months in fridge (2 to 8°C), in freezer (−20°C), and with autoclaved antimicrobials, had significant inhibition zones. The autoclaved fermentation extracts also had antimicrobial activity, however, with reduced inhibition zones (Figure 3). These compound produced by isolate AR18 demonstrated having greater stability faced with different treatments, showing larger inhibition zones, as can be seen in Figure 3.

## 4. Discussion

Direct cloning of fragments with high metagenomic DNA molecular weight, as was employed in this study with the construction of the library with fosmid vectors, constitutes an excellent tool to obtain and prospect metagenomic DNA from soils [13, 23]. However, the advantages of using metagenomics when searching for secondary metabolites run into the limitations of this technique to prospect and isolate these substances. The success of this technology depends both on a capacity to clone contiguous fragments of DNA in appropriate vectors and the capacity of the vector to express heterologous DNA [24], enabling the detection of the compound of interest by the screening methods.

Limitations of the metagenomic technique to the use of metagenomics in search of antimicrobials must have contributed to the results found in this work, in which we used two methodologies to prospect antimicrobials using soil microorganisms. With metagenomics, although it enables us to access a larger diversity of microorganisms, we did not detect antimicrobial production by any of the 2688 clones of either soil samples. But with the classic Actinobacteria isolation approach we obtained antimicrobial production in 2 of the 24 isolates from Atlantic Rainforest soil (Figures 1 and 2, Table 1). Probably due to technical limits, the metagenomic approach has not been successful in the discovery and development of new antibiotics [10, 25]. Our group has already had good results with the use of metagenomics to prospect enzymes [26], but we still have not had positive results in prospecting antimicrobial substances.

Some authors have highlighted that the use of* E. coli* as host in metagenomic libraries for heterologous expression limits the DNA expression of many soil microorganisms [27, 28]. According to Gabor et al. [28], the actinomycetes are the group least likely to have an expression in* E. Coli*, only 7% of the genes, which is quite relevant when the objective is the search for antimicrobial agents, due to the importance of this group in the production of these compounds. Another problem can occur due to many of uncultivable microorganisms not having the capacity to produce antimicrobials; due to the size of the genome, many of these organisms are members of groups with relatively small genomes, so they would be unable to produce such compounds [10, 29].

These results strengthen the importance of getting back to research with microorganisms already recognized for producing antimicrobials, such as Actinobacteria. Brazilian tropical soils have a large microbiological diversity and are especially rich in bacteria of the genus* Streptomyces sp. *[30], the largest producers of antimicrobials known. However, few research works are still aimed at prospecting antimicrobial substances. Some of the research works have found substances of industrial importance, such as enzymes [16, 31] and antimicrobials that act against pathogenic microorganisms [15, 32] and also against microorganisms involved in biocorrosion processes in the oil industry [17].

One of the challenges in research of antimicrobial substances from soil Actinobacteria is the isolation of a really new compound that acts against resistant microorganisms, which is today's biggest therapeutic concern. To work around the problem of isolating already known substances, we sought the isolation of microorganisms in an untapped source (the Atlantic Rainforest) and screened for antimicrobial activity against a microorganism already known for its resistance to clinical drugs (methicillin-resistant* S. aureus*). Some research works have even used microorganisms strains such as* E. coli* with insertion of antibiotic resistance genes [33]. These approaches can help select new compounds [34] that are effective against resistant pathogens. The inhibition results of antimicrobials against methicillin-resistant* S. aureus *(MRSA) are important because since it is a multidrug-resistant microorganism, the crude extracts of both AR5 and AR18 isolates presented significant inhibition halos (Figure 2). The MRSA strains are one of the major causes of hospital infections that are becoming increasingly difficult to fight due to resistance to various drugs [35].

The isolates from the planting system did not present inhibition against the microorganisms tested in this work; however, these isolates can have potential for the production of antimicrobials against other untested microorganisms, or still, produce antifungal compounds that have not been studied in this work. It will be necessary to conduct inhibition tests against other important pathogens and against filamentous fungi in order to better exploit the potential of these isolates.

Both of the antimicrobials researched in this work demonstrated good stability regarding storage time and different temperatures, having even been resistant to autoclaving (Figure 3). These results are important for enabling, for example, stocking the compounds for later studies, such as liquid chromatography. Some known antimicrobials, such as chloramphenicol, aminoglycosides, and quinolones [36], have already been characterized as thermostable, and even so, not all are stable in autoclaving. Thermal stability is an important characteristic, because it enables the antimicrobial to be used in industrial applications with high variation in temperature [37, 38].

The antimicrobial producing isolates (AR5 and AR18) have a morphology characteristic of Actinobacterias of the genus* Streptomyces sp. *[21], such as the production of white mycelium that gains a grayish color as the colony ages, and under the microscope, they display thin hyphae and spore chains. These isolates are phylogenetically characterized with sequencing of 16S rDNA, as well as morphological and physiological observations; it has been possible to classify both isolates as belonging to the* Streptomyces sp.* genus (data not shown).

In view of the history of antimicrobials discovery and development, and due to the unfavorable considerations regarding the employment of metagenomics for this purpose, getting back to research on isolating microorganisms from environment samples, especially Actinobacteria, is still a good alternative for prospecting these substances. However, it is necessary to search for antimicrobials capable of acting against the many microorganisms resistant to clinical drugs. Research works exploiting preserved environments or those that have not been accessed, such as the present work, are promising, because they can discover new compounds, with new targets and action mechanisms, and can be the solution to the fight against growing microbial resistance.

## 5. Conclusions

In the present work, metagenomics did not show good results in the search for antimicrobials, probably because of limitations of the metagenomic technique. The classic method of isolating soil Actinobacteria demonstrated that it continues to be a promising approach, with which 2 of the 24 isolates of Atlantic Rainforest soil produced antimicrobials. The Atlantic Rainforest is a promising place for the isolation of substances that are important to the industry, such as antimicrobial compounds, and has a rich microbial biodiversity that has been virtually unexploited for this purpose. Both of the antimicrobials isolated in this work (AR5 and AR18) had thermal stability when stored in different temperatures and after autoclaving. Both compounds are promising, especially because of their action against* S. aureus* MRSA, an important multidrug-resistant human pathogen. These antimicrobials will be studied with the use of techniques that enable us to elucidate the chemical structure of the compounds, such as infrared spectroscopy, mass spectrometry, and nuclear magnetic resonance.

## Figures and Tables

**Table 1:** Inhibition diameters presented by the two Actinobacteria isolates from Atlantic Rainforest soil.

Test organisms	Number of isolated/sample	Zone of inhibition (mm)
*S. aureus* CCMB 262	5-Atlantic Rainforest soil (AR5)	24
*S. aureus* CCMB 262	18-Atlantic Rainforest soil (AR18)	28
*S. aureus* MRSA	5-Atlantic Rainforest soil (AR5)	16
*S. aureus* MRSA	18-Atlantic Rainforest soil (AR18)	18
